# Trajectory of skill acquisition, loss, and regain in females with classic Rett syndrome

**DOI:** 10.21203/rs.3.rs-8245192/v1

**Published:** 2025-12-15

**Authors:** Jeffrey L. Neul, Timothy A. Benke, Eric D. Marsh, Sarika U. Peters, Cary Fu, Jonathan K. Merritt, Alan K. Percy

**Affiliations:** Vanderbilt University Medical Center; Children’s Hospital of Colorado and University of Colorado; University of Pennsylvania Perelman School of Medicine and Children’s Hospital of Philadelphia; Vanderbilt University Medical Center; Vanderbilt University Medical Center; Vanderbilt University Medical Center; University of Alabama at Birmingham

**Keywords:** Rett syndrome, Developmental milestones, Gain/Loss/Regain, Natural History Study, Clinical trial endpoint

## Abstract

**Background:**

To characterize frequency, timing, and trajectory of gain, loss, and regain of developmental skills in Classic Rett syndrome (RTT).

**Methods:**

The frequency and timing of gain, loss, and regain of 51 developmental skills from 1228 females with Classic RTT and a pathogenic loss-of-function variant in *MECP2* was assessed during in-person visits from participants enrolled in the US Natural History Study. The percentage of participants experiencing gain, loss, or regain events, mean and median age of event and time to event with confidence intervals, and the cumulative incidence curves were calculated and compared to normative data using SPSS v29.0.2.0. One-year incidence of either gain or regain of each skill from 0–20 years old and one-year incidence of either gain or regain of any of 51 developmental skills was calculated.

**Results:**

The acquisition of skills was greatest for lower-level skills and conversely lowest for more advanced skills. Acquisition of skills peaked at 6 years. Skill loss occurred mainly within 2 years of acquisition. Loss of fine motor, communication, and social adaptive skills changed little after age 6 years. The regain of lost skills involved less than 30% of fine motor, communication, and social adaptation. Regain of skills generally ceased by age 6 years.

**Conclusion:**

These results provide critical endpoints essential for conducting clinical trials in RTT. The lack of acquisition of skills beyond age 6 years and absence of loss or regain of previously lost skills, aside from gross motor features, beyond 6 years suggest that functional gains in these realms would represent important indicators of efficacy.

## Introduction

Rett syndrome (RTT) is a rare neurodevelopmental disorder primarily caused by pathogenic variants in *Methyl-CpG-Binding Protein 2* (*MECP2*) [1, 2]. RTT primarily, but not exclusively, affects females [3, 4] and has a characteristic disease pattern with “normal” initial development, followed by developmental delay and then regression with loss of hand skills and spoken language, gait problems, and hand stereotypies [5, 6]. After regression, a “Pseudostationary” stage occurs that has been characterized by no further skill loss and potential skill regain [7, 8]. Eventually, a “Late Motor Decline” stage occurs, characterized by muscular rigidity, declining gross motor ability, and onset of Parkinsonian features [7, 9–11].

Although previous work has described aspects of the gain and loss of developmental skills (also referred to as “developmental milestones”) [12–15], data on the frequency and timing of skill gain and loss remains incomplete, and evaluation of skill regain is minimally characterized. The need for this information is driven by the demonstration that restoration of MeCP2 function, even after symptom onset, can reverse phenotypic problems in RTT mouse models [16, 17]. This provided the foundation for disease modifying therapy development, such as gene therapy clinical trials for RTT [18]. Transformative therapies for RTT should change the course of the disease by enabling the acquisition of new skills, restoring lost skills, or preventing skill loss. Therefore, it is critical to understand the expected trajectory of skill gain, loss, and regain in RTT to objectively evaluate the therapeutic impact of novel therapies compared to historical data.

From 2006–2021, the multi-site US Rett syndrome and Rett-related disorders Natural History Study (RNHS) longitudinally evaluated a large number of people with RTT [19], providing information on the clinical features and disease progression of RTT [20–28], identifying and evaluating putative biomarkers [29–33], developing outcome measures [34–40], and establishing a clinical research network to enable the development of the first FDA-approved treatment for RTT [41]. The RNHS captured information on developmental skill gain, loss, and regain from people with RTT, and analysis of early data from the RNHS provided information about developmental skill gain and loss [13]. However, this previous work was limited by a smaller sample size and data on fewer skills compared with information available after the RNHS completion. Further, the previous work focused on skill gain or loss and did not evaluate skill regain or the incidence of skill gain or regain within a clinical trial timeframe.

To address this need, we characterized the frequency and timing of skill gain, loss, and regain in females with Classic RTT and a pathogenic variant in *MECP2* using the extensive RNHS dataset. We evaluated the time from gain to loss and the time from loss to regain to understand the temporal relationship between events. Further, we characterized the fraction of participants who gained a skill beyond either 4 or 6 years old, regained a skill beyond 6 years old or more than 2 years after loss, as well as determining the one-year incidence of gaining or regaining a skill. This work provides detailed information on the trajectory of developmental skill gain, loss, and regain in RTT to facilitate earlier diagnosis, guide clinical management, and establish foundational information relevant to the evaluation of disease modifying therapies for RTT.

## Methods

### Participants

Participants were enrolled in the Rett syndrome and RTT-related Disorders Natural History Study (RNHS, NCT00299312, NCT02738281), a longitudinal study incorporating caregiver-provided historical and clinically observed information spanning from 2006 to 2021 [19]. Participants were included in the RNHS if they had a clinical diagnosis of RTT based on the clinical consensus criteria [6], had a pathogenic variant in *MECP2* but did not meet RTT clinical diagnostic criteria, or had a diagnosis of a RTT-related disorder (*MECP2* duplication syndrome, *CDKL5* deficiency disorder, or *FOXG1* syndrome). Participants enrolled provided genetic testing results, although not all enrolled participants had an identified pathogenic genetic variant. All participants or their legally authorized representatives provided informed consent before participation in the RNHS. The RNHS was conducted in accordance with the Declaration of Helsinki, and approved by the University of Alabama, Birmingham Institutional Review Board. A Certificate of Confidentiality was provided by the National Institute of Child Health and Development (NICHD). Overall, 1826 individuals participated in the RNHS with an average of ~ 5 visits per individual.

For this study, we restricted analyses to female participants diagnosed with Classic RTT with a pathogenic variant in *MECP2* who had some data collected on developmental milestone skill gain, loss, or regain (N = 1228). The mean age of first visit for this cohort was 11.7 years (Standard Error of the Mean [SEM] = 0.28 years), with a median age of enrollment of 8.2 years (Interquartile Range [IQR] = 4.2–16.4 years). The median number of study visits was 5 (range: 1–17; IQR: 2–8). The majority (71.3%) were white and not Hispanic (see **Additional file 1: Table S1 Demographic Information** for complete demographic information). **Additional file 2: Table S2 MECP2 Mutation Frequency** displays the breakdown of the frequency of common *MECP2* point mutations and other mutation groups in the analysis cohort. For *MECP2* mutation groups, we grouped rare point mutations affecting the same amino acid codon as common recurrent point mutations into the common point mutations. Specifically, participants with R106Q variants were grouped with R106W; those with R133P or R134C variants were grouped with R133C; participants with T158A or T158P were grouped with T158M, and participants with R306H or R306L were grouped with R306C. The Early Truncations group included all nonsense of frameshift variants before position R270, including splice site variants, but excluding the common recurrent nonsense point variants (R168X, R255X, R270X). The C-terminal Truncations group included all frameshift or nonsense variants after position R270, excluding the common recurrent nonsense point mutation R294X. Other point mutations encompass all other missense variants aside from the common recurrent variants (R106W, R133C, T158M, R306C). This approach to grouping pathogenic genetic variants is consistent with previous work [42, 43].

### Visit Schedule and Assessments

Participants were assessed in a structured in-person clinical research visit (lasting ~ 1–2 hours), which occurred longitudinally at pre-defined intervals based on age of enrollment, ranging from twice per year to every other year [19]. In-person evaluations utilizing structured research forms including caregiver completed history and assessment forms and questionnaires, clinical histories, structured clinical exams, and clinician-completed rating scales. Information on the gain, loss, and regain of developmental skills was captured through a detailed, direct interview with caregivers. Caregivers were provided a developmental skill history questionnaire prior to the baseline visit and asked to provide information on whether a specified skill was gained, lost, or regained and the age of gain, loss, or regain (or indicate age of skill event being unknown). Caregivers were instructed to review and refer to aids to improve recall such as baby books and pictures, and review of prior medical evaluations by primary care physicians and any subspecialists. During the in-person baseline visit, the developmental skill history questionnaire was reviewed by the clinical investigator with the caregiver to discuss and confirm the answers provided by the caregiver, as well as raise questions about the caregiver’s responses with regards to the current ability of the participant (*e.g*. if caregiver stated that the participant had gained but not lost a skill, but the participant was not able to perform the skill during the clinical evaluation or by parent report, the investigator would ask for clarification of the developmental history report provided that indicated that the skill was not lost). At all subsequent visits, the developmental history log was reviewed with the caregiver to assess whether any changes occurred since the previous study visit with regards to skills being gained, lost, or regained and updates to the form made to reflect these changes.

From 2006–2014, the developmental skill history questionnaire queried 31 developmental skills (NCT00299312, RNHS protocol 5201). In 2015, upon initiation of the third funding cycle, the data capture forms underwent a major revision including modification of the developmental skill history questionnaire (NCT02738281, RNHS protocol 5211), which added a number of new developmental skills to expand the range and detail of developmental milestones assessed and dropped three skills captured in the previous protocol (Roll Back to Front, Pedal Tricycle, and Stopped Being Visually Attentive) based on analysis showing either floor or ceiling effects for these three skills. The new developmental skill history questionnaire included a total of 51 developmental skills, with 28 mapping to skills captured in 5201 and the addition of 23 additional skills. The names of the skills in both protocols, the mapping of the skills between the two protocols, and the names used for the skills in this paper are provided in **Additional file 3: Table S3 Developmental skill mapping between protocols 5201 and 5211**. For this study, we analyzed the 51 skills assessed in protocol 5211 (2015–2021) and merged the information from the two protocols as shown in **Additional file 3: Table S3 Developmental skill mapping between protocols 5201 and 5211**. For participants meeting the inclusion criteria for these analyses defined above (N = 1228), 586 participated solely in 5201, 307 participated only in 5211, and 335 participated in both 5201 and 5211. For participants evaluated in both 5201 and 5211, initial age of skill events provided were used in cases when there were discrepancies between dates identified between data collected in the two protocols; however, updated information collected during 5211 reflecting changes in skill event occurrence from 5201 were used to reflect changes in skill gain, loss, or regain over time. Total Ns (Tables 1–3) reflect total number of participants from 5201 and or 5211 with information available on skill gain, loss, or regain post data processing and cleaning described below. The calculation of the mean and median age of skill gain, loss, or regain was based only data for which a specific age of event was obtained (excluding those participants with an “age unknown” indicator), thus the total number of participants included in these calculations are lower than the total number with data indicating whether the skill event occurred or did not occur.

### Data processing and cleaning prior to analyses

Prior to analysis, the raw data went through a series of processing and data cleaning steps. Prior to data cleaning, data fields coded as “Yes” or “No” were recoded as “1” or “0”. The full description of the cleaning process criteria is provided in **Additional file 4: Supplemental Methods**, including the number of changes (n) made according to each criterion. Overall, this process resulted in changes to 26,170 data cells, which represents changes to 4.64% of all data cells. The changes fell into the following broad categories:

1. Most changes were to skill event occurrence indicators (n = 23,575; 4.22%), with the vast majority of these removing subsequent skill event indicator of “No” when preceding skill event did not occur (n = 22,537, 4.0%). The remaining changes to skill event occurrence indicators (n = 1,221; 0.22%) added missing skill event indicators to subsequent skill events when preceding skill event occurred, correcting preceding skill event indicators when subsequent skill occurred, or adding missing skill event occurrence indicator when age of skill event was provided or age of skill event was indicated as “unknown”.
2. The next largest group of changes were corrections to age of skill event unknown indicators to remove the age of skill event unknown indicator if the age of skill event was provided, or add skill event unknown indicator when event occurred but the age of skill event was not entered and the age of skill event unknown indicator was blank (n = 1,205, 0.2%)
3. Correcting logical age inconsistencies (*e.g*. the age of skill event entered was beyond the age of last visit or before the preceding skill event age) was the next largest group of changes (n = 736, 0.13%)
4. Identifying entries for the age of skill gain that was at an unrealistically young age of gain and removing age of gain and adding age of gain unknown indicator was the next largest group of changes (n = 590, 0.1%)
5. Finally, the smallest group of changes were based on review of source data (n = 64, 0.01%)

After cleaning, all ages were converted from “months” to “years” (with 1 decimal point) by dividing months by 12. For all skill events that were identified as not having occurred (and not blank), the age of last visit (in years) was inserted into the age of event field as “censored age” data for subsequent survival analysis.

### Statistical Analyses and Data Visualization

All data was stored and processed in Excel v16 (Microsoft). The presentation of the data for this study is descriptive; no hypothesis tests were performed. SPSS v29.0.2.0 (IBM, Armon, NY, USA) was used to calculate proportions, mean values with standard error of the mean (SEM), median values, and Kaplan-Meyer (cumulative incidence) survival tables. The overall rate of gain, loss, or regain for each skill for all cases with information on the status of the event (including those without information on the age of the event) was calculated as the proportion (presented as percentage) of individuals in which the event occurred over all individuals with information for that skill event. Because the denominator varies across skill events, the raw number for the numerator (individuals in which the skill event occurred) and the denominator is presented. Confidence Intervals (95%) for skill event proportions were calculated using the BINOMIAL function in SPSS using Likelihood (normal approximation) unless the number of individuals in which an event occurred [n(p)] or did not occur [n(1-p)] was less than 5, or if the proportion of occurrence (p) or non-occurrence (1-p) was less than 0.10, in which the Clopper-Pearson exact method was used. Mean and median age of event was calculated using only individuals with available information as no imputation was performed. Median skill event age and age range (5–95%) was calculated using the ROUND function in SPSS. Cumulative incidence tables were calculated using Kaplan-Meyer survival analysis, with censoring if the skill event did not occur. The age of last visit was used for the censored age for skill gain, loss, or regain. For time from gain to loss or time from loss to regain, the censored age was the age of last visit minus the age of the preceding skill event. The cumulative incidence tables were used to calculate the percent of individuals who gained a skill beyond 4 years or 6 years of age, regained a skill beyond 6 years of age, or regained a skill more than 2 years after losing that skill. Additionally, we used these cumulative incidence tables to determine the percentage of individuals who did not gain a skill by the age the US Centers for Disease Control (CDC) indicates that most children (75% based on normative data) are expected to achieve that skill [44]. This calculation was only done for those skills for which CDC normative age recommendations for surveillance are available (41 of the 51 skills assessed in this study). Graphical representation of cumulative incidence curves (survival or 1-survival curves) was generated using R v4.5 (www.r-project.org), with Powerpoint v16.1 (Microsoft) used to create merged figures of representative cumulative incidence curves.

The incidence of either gaining or regaining a skill during a one-year interval was calculated for one-year bins from 0–20 years of age. For the analysis of one-year interval incidence, information of gain or regain of skills beyond 20 years of age was not included. To determine the one-year incidence of gain or regain for each skill, the number of individuals who gained or regained that skill within the one-year interval was divided by the total number of individuals eligible to either gain or regain the skill during the specified interval. The detailed logic used to determine skill gain or regain and eligibility in each interval is provided in the **Additional file 4: Supplemental Methods**. For each individual, the total number of skills gained or regained in each one-year interval was calculated, and the percentage of individuals who gained at least 1 of 51 or 1 of the 38 skills selected (described in the results section) was determined by dividing by the total number of participants eligible to gain or regain a skill in that interval.

## Results

### Developmental Skill Gain

Delayed or absent acquisition of developmental skills is a well-recognized feature in RTT [12–15]. The percentage of participants in this study who gained specific developmental skills ranged from 99.5% for Social Smile to 2.4% for Shared Stories (Table 1). Across developmental skill domains (gross motor, fine motor, receptive language, expressive language, social/adaptive skills), the frequency of skill gain was highest for lower level and earlier acquired skills than for more advanced skills. For example, within the gross motor skill domain, nearly 100% of participants gained early skills such as lifting head, rolling from tummy, and sitting with or without support, but only ~ 60% gained the ability to walk independently (Table 1). Similarly, within expressive language, nearly all participants gained early skills such as Cooing and Babbling, but less than 25% gained Spoken Phrases. Out of the 51 skills characterized, 21 individual skills were gained by over 80% of all participants and only 2 skills were gained by less than 20% of all participants (Down Stairs Without Help and Shared Stories).

**Figure 1:**
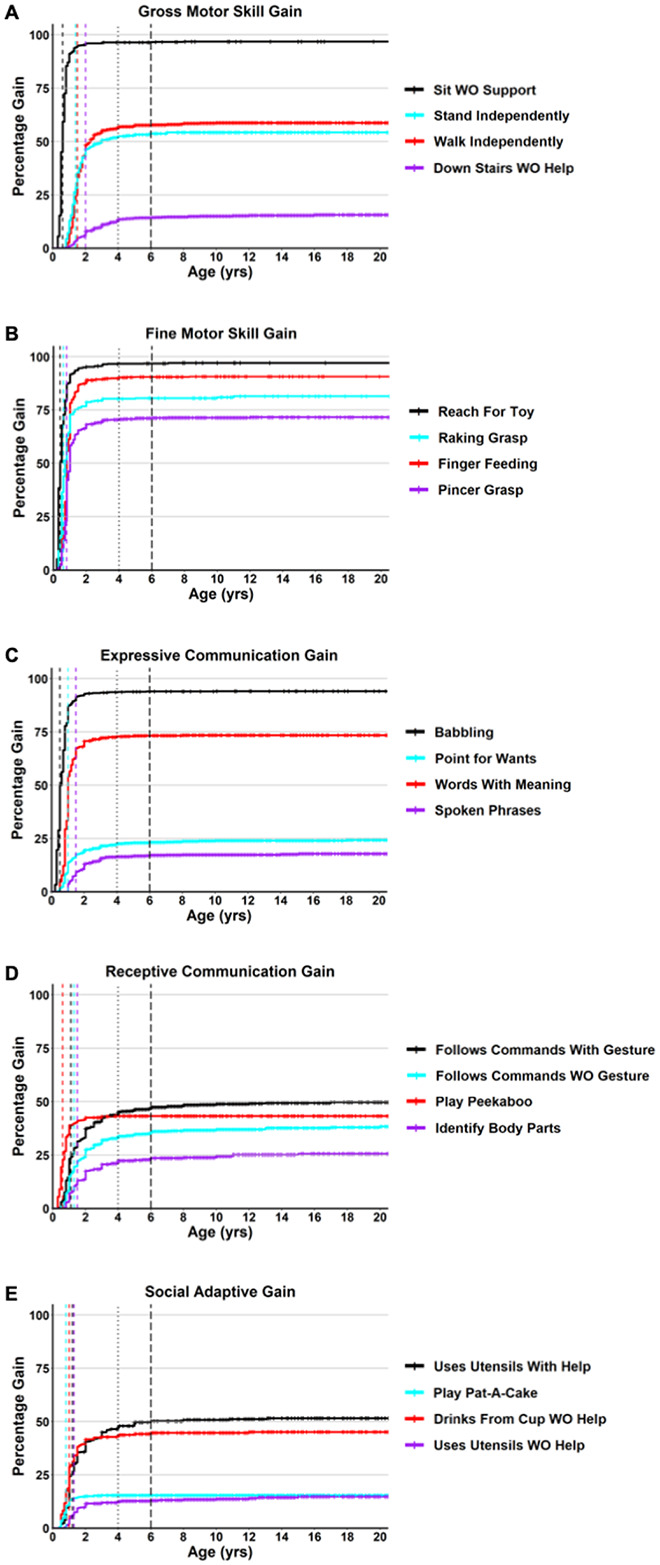
Cumulative incident of gain for representative skills. Cumulative incidence curves for age of skill gain are shown for skills representing gross motor (A), fine motor (B), expressive communication (C), receptive communication (D), and social/adaptive (E) skill domains. The colors for each representative skill are shown in the legend, with censored data points shown as cross lines, a colored vertical line for the median age of gain for that specific skill, wide spaced dashed vertical line is at 4 years of age and closely spaced dashed vertical line is at 6 years of age in all graphs.

In the participants that age of skill gain was known, the mean age of skill gain (Table 1) ranged from 0.2 years (Like Being Held) to 6.9 years (Shared Stories), and the median ages of skill gain ranged from 0.1 years (Like Being Held) to 3 years (Point to 1 Color). Examples of cumulative incidence curves (Kaplan-Meyer 1-Survival curves) for the gain of representative developmental skills are shown in Fig. 1, with curves for all developmental skills provided in **Additional file 5: Figure S1**. The median age of skill gain was one year or younger for 33 of the 51 skills (65%), and two years or younger for 50 skills (98%) (Table 1). For 26 of the 51 developmental skills (51%), 95% of participants who gained that skill did so by 2 years old (Table 1). By 4 years of age, this increased to 73% of the skills (37 of 51 skills), and by 6 years old it increased to 92% of the skills (47 of 51 skills). For each developmental skill, we calculated the percentage of participants who gained the skill after age 4 or 6 years of age (Table 1). The percentage of participants who gained a specific skill after 4 years of age was less than 5% for all skills except Point to 1 Color. The percentage of participants who gained a specific skill after 6 years of age was less than 2.5% for 50 of the 51 skills, with the majority (35 of 51) of the skills being gained by 1% or less of participants who gained the skill. These data demonstrate that acquisition of the skills evaluated in this study generally occurs early in life in people with RTT, with the majority (95%) of people who gain a specific skill doing so before 4 years of life, and 97.5–99% who gain a specific skill doing so before 6 years of life.

The US Centers for Disease Control (CDC) has developed normative guidelines for developmental surveillance based on the age that most (> = 75%) of children should attain specific developmental skills to help with early identification of children with developmental delays or disabilities [44] (https://www.cdc.gov/act-early/index.html). Of the 51 developmental skills evaluated in this study, CDC age-specific surveillance recommendations are available for 41 skills. We characterized the percentage of participants in this study who had not yet gained a specific skill by these CDC recommendations (Table 1). The percentage of participants who did not gain a skill by the CDC guidelines ranged from 14.6% (Sit Without Support) to 99.5% (Shared Stories). Notably, in 26 of the 41 (63.4%) skills with CDC recommended age of gain, the majority (> 50%) of study participants did not gain the skill by the CDC threshold age. For advanced skills such as Shared Stories, the age that most children are expected to gain the skill is beyond the typical age of regression in RTT (~ 18 months [12, 45]). However, it is notable that a significant percentage of participants fail to acquire lower-level skills that are expected to be gained before 6 months of life, such as Lift Head (59.3%), Hold Bottle (84.8%), Social Smile (45.9%), and Eyes Fix and Follow (70.2%). Furthermore, a significant percentage also failed to gain major milestones that are expected to be gained by 18 months of life (1.5 years) such as Pull to Stand (62.1%), Walk Independently (66.8%), Pincer Grasp (41.6%), Words with Meaning (45.8%), and Point for Wants (84.2%). As the average age of diagnosis of RTT is 2.7 years [46], these notable early departures from expected age of gain of developmental skills should raise concerns for caregivers and primary care providers.

### Developmental skill loss

Regression, or loss of previously acquired developmental skills, is a defining characteristic of RTT [5, 6]. Therefore, we calculated the percentage of participants who lost specific skills previously gained, as well as the mean and median age of skill loss (Table 2). The percentage of participants who lost previously gained skills ranged from 6.6% (Lift Head) to 88.6% (Words with Meaning). The high frequency of loss of Fine Motor and Expressive Language skills is consistent with regression in these domains being a *conditio sine qua non* of RTT [15]. However, Gross Motor, Receptive Language, and Social/Adaptive skills are also lost in a significant fraction of people with RTT.

**Figure 2:**
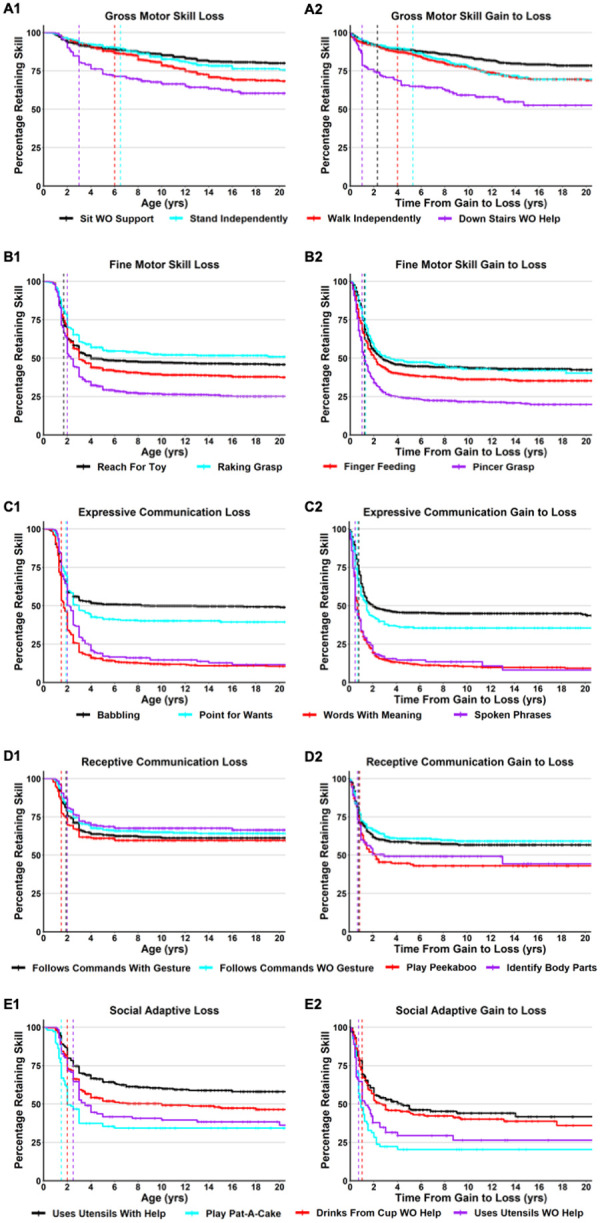
Cumulative incident of loss and time from gain to loss for representative skills. Cumulative incidence curves for age of skill loss are shown for skills representing gross motor (A), fine motor (B), expressive communication (C), receptive communication (D), and social/adaptive (E) skill domains. The age of skill loss is displayed in panels A1, B1, C1, D1, and E1, colors for each representative skill are shown in the legend, with censored data points shown as cross lines, a colored vertical line for the median age of loss for that specific skill. The time from gain to loss is displayed in panels A2, B2, C2, D2, and E2, with a colored vertical line for the median time of gain to loss for that particular skill.

The mean age of skill loss ranged from 1.6 years old (Cooing, Respond to Sounds) to 8.2 years old (Down Stairs With Help), and the median age of loss ranged from 1.3 years old (Cooing) to 7.3 years old (Down Stairs With Help). The median age of skill loss was 3 years old or less for 46 of the 51 developmental skills (90%), and in 31 developmental skills, 95% of participants lost skills by 6 years old. Cumulative incidence curves (Kaplan-Meyer Survival curves) of loss of representative developmental skills are shown in Fig. 2 (Panels A1, B1, C1, D1, E1), with loss curves for all developmental skills provided in Additional file 6: **Figure S2**. Notably, while most skill domains show a relatively tight window of loss across all participants, the loss of Gross Motor domain skills such as Stand Independently or Walk Independently occurs more gradually and over a longer time period (Table 2 and Fig. 2A1).

Given the variation in the age of skill gain, we calculated the time from gain to loss for all developmental skills (Table 2). The mean time from gain to loss ranged from 0.7 years (Point to 1 Color) to 6.6 years (Stand Independently), and the median time from gain to loss ranged from 0.5 years (Words With Meaning, Spoken Phrases) to 5.3 years (Stand Independently). Overall, the median time from gain to loss was less than 2 years for 44 of the 51 developmental skills (86.3%). Consistent with the analysis of the age of loss, the skills that had median time from gain to loss greater than 2 years were all within the gross motor domain such as Stand Independently (5.3 years), Walk Independently (4.0 years), or Down Stairs With Help (5.0 years). Figure 2 (Panels A2, B2, C2, D2, E2) shows cumulative incidence curves for the time from gain to loss for representative developmental skills, with gain to loss cumulative incidence curves for all developmental skills provided in **Additional file: Figure S3**.

### Regain of developmental skills

**Figure 3:**
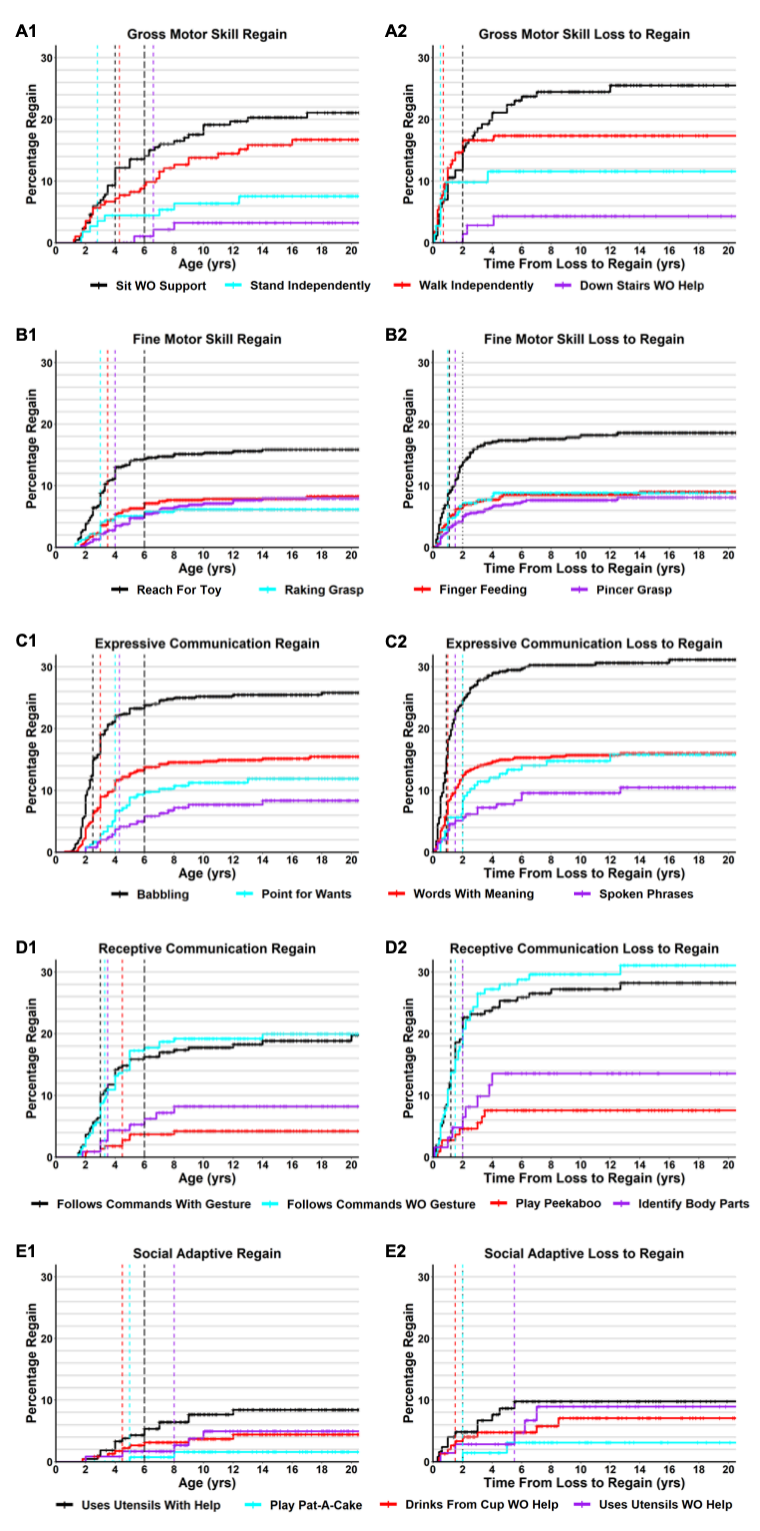
Cumulative incident of regain and time from loss to regain for representative skills. Cumulative incidence curves for age of skill regain or time from loss to regain are shown for skills representing gross motor (A), fine motor (B), expressive communication (C), receptive communication (D), and social/adaptive (E) skill domains. The age of skill regain is displayed in panels A1, B1, C1, D1, and E1, with colors for each representative skill shown in the legend, censored data points are shown as cross lines, a colored vertical line for the median age of regain for that specific skill, and a closely-spaced black dashed line demarking 6 years of age in all graphs. The time from loss to regain is displayed in panels A2, B2, C2, D2, and E2, with a colored vertical line for the median time of loss to regain for that particular skill, and a closely spaced black vertical line demarking 2 years from loss to regain in all graphs.

While developmental delay and regression are well known features of RTT, it has been recognized that there are instances of regain of previously lost developmental skills in RTT, primarily in the Gross Motor domain [47–50]. For the developmental skills captured, we characterized the percentage of participants who regained each skill and age regained (Table 3). The range of percentage of regain of individual skills ranged from 2.1% (Play Pat-A-Cake) to 80.6% (Social Smile). For the majority of skills (31 of 51, 60.8%), the percentage of participants who lost and regained that skill was under 20%. In general, low level social and receptive language skills (such as Social Smile, Desire Social Attention, Respond to Own Name) were skills with the highest percentage of regain amongst participants, whereas a low percentage of participants regained higher level skills. The mean age of regaining skills ranged from 2.7 years old (Respond to Sounds) to 8.0 years old (Up Stairs With Help), and the median ranged from 2.0 years old (Respond to Sounds, Attention to Loud Sounds) to 9.0 years old (Up Stairs With Help). Of the 51 developmental skills regained, 46 (90.2%) had a median age of regain under 6 years of age. The percentage of individuals who lost a specific skill and then regained that skill after 6 years of age was under 10% (out of all who lost the skill) for all 51 skills, and 5% or less for 40 of the 51 skills (78.4%). Cumulative incidence curves (Kaplan-Meyer 1-Survival curves) of regain of representative developmental skills are shown in Fig. 3, with regain curves for all developmental skills provided in **Additional file 8: Figure S4**. Broadly, while there is evidence of skill regain after loss in RTT, the frequency is generally low and typically occurs before 6 years of age.

To account for variation in the age of loss for specific skills (Table 2), we calculated the time from loss to regain for each developmental skill (Table 3). The mean time from loss to regain ranged from 0.9 years (Stand Independently, Walk Independently, Ran 10 Feet) to 4.1 years (Uses Utensils Without Help), and the median time from loss to regain ranged from 0.5 years (Stand Independently, Up Stairs Without Help) to 5.5 years (Uses Utensils Without Help). Nearly all skills had a median time from loss to regain of 2 years or less (46 of 51, 90.2%). For 35 of the 51 developmental skills (68.6%), the percentage of participants who regained that skill (out of all who lost that skill) beyond 2 years from loss was less than 10%, and for 25 of the 51 skills this percentage was less than 5%. Notably, skills with a low percentage of regain beyond 2 years from loss include all the fine motor hand skills (Ranging from 1.5% for Turning Pages in Book to 4.8% for Reach For Toy), more advanced expressive verbal skills such as Words with Meaning (3.5%) or Spoken Phrases (4.9%), and higher-level gross motor skills such as Stand Independently (1.7%) or Walk Independently (0.7%), with the exception of Up Stairs With Help or Down Stairs With Help. In contrast, lower-level skills and non-verbal Receptive skills had a higher percentage of regain beyond 2 years from loss. Cumulative incidence curves (Kaplan-Meyer 1-Survival curves) of the time from loss to regain for representative developmental skills are shown in Fig. 3, with loss to regain curves for all developmental skills provided in **Additional file 9: Figure S5**. Overall, when skills are regained after loss, for the majority of skills this regain occurs within the first 2 years after loss.

### Incidence developmental skill gain or regain in one-year intervals

**Figure 4:**
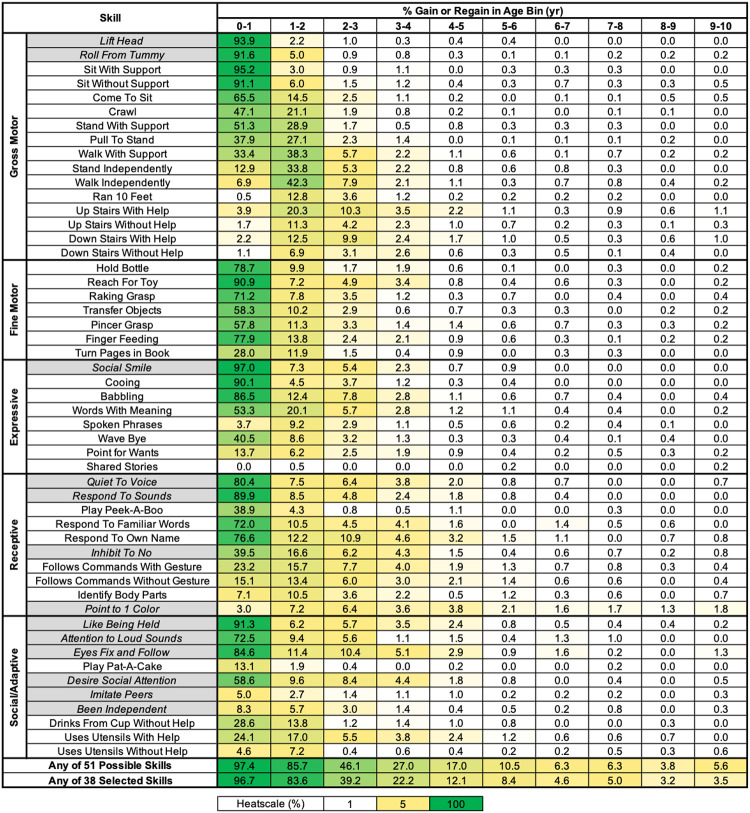
One-year interval incidence of skill gain/regain for individual skills and for any one skill. The percentage (%) of participants eligible within one-year age intervals who gained or regained a specific skill is shown, as well as the percentage of eligible participants within the age bin who gained at least 1 skill out of all 51 skills characterized or at least 1 skill out of a restricted subset of 38 skills. Grey highlighted skills identify those skills not included in the restricted subset of 38 skills. The heatmap color coding shown at the bottom, with white for 0%, yellow for 5%, and dark green for 100%.

Because we observed that developmental skill gain, loss, or regain in RTT primarily occurs within the first couple years of life for the majority of skills (Tables 1–3), we were interested in evaluating the incidence of either gaining or regaining a developmental skill in one-year intervals spanning from 0–20 years old. Figure 4 displays the percentage of eligible participants in each one-year interval who gained or regained a specific developmental skill from age 1–10 years old. We found that the frequency of gain or regain for any of the skills drops to < 2% per year after age 10 (**Additional file 9: Figure S6** for results spanning the entire age range). For all skills, the one-year incidence of gain or regain is below 4% after 4 years of age. After age 6, the one-year incidence of gain or regain is less than 1% per year for all skills except for Responds To Familiar Words, Responds to Own Name, Point to 1 Color, or Eyes Fix and Follow.

We calculated the frequency of gaining or regaining one or more of any of the 51 skills during one-year intervals (Fig. 4). There is a sharp drop-off in frequency with increasing age, with the overall percentage of eligible participants who gain or regain at least 1 of any of the 51 skills dropping below 6.3% per year after 6 years of life. While this demonstrates that after a given age of life the likelihood of gaining or regaining even one skill becomes unlikely, we were interested if selecting a more limited set of developmental skills would further decrease this likelihood. To evaluate this, we performed a similar analysis on a restricted set of developmental skills, removing early acquired skills for which attainment (either gain or regain) might have limited functional meaningfulness or may have been difficult to clearly assess (*e.g*. Lift Head, Quiet to Voice, Respond to Sounds, Social Smile, Like Being Held) or skills with a high rate of gain or regain beyond 3–4 years old (*e.g*. Point to 1 Color). The thirteen skills removed from this restricted analysis are indicated by grey highlights in Fig. 4, leaving the remaining 38 skills for analysis. In this restricted set of developmental skills, the percentage of participants who gained at least one of these 38 skills fell below 5% per year after 6 years of life. This demonstrates that further restriction of which developmental skills to include in an analysis of the one-year incidence of gain or regain of one or more skills, based on consideration of meaningfulness and impact of acquiring a skill on the affected individual, could further decrease the one-year frequency of gain or regain of any of the selected skills.

## Discussion

Alteration of the developmental trajectory of psychomotor skills (“developmental milestones”) is a major feature of RTT [5], typically described as “apparently normal” development in early infancy, followed by developmental delay, subsequent regression of previously acquired skills, and then stabilization with some regain of previously lost skills. Previous studies [12–14, 51, 52] primarily presented the proportion of individuals with RTT that gain or lose a skill with limited quantitative information on the timing of skill gain or loss or the proportion and timing of regaining previously lost skills. These gaps reflect the challenge of gathering sufficient data in rare disorders such as RTT. To address these gaps, we analyzed data on developmental skill trajectory in a large cohort (n = 1228) of females with Classic RTT collected over the sixteen years from the US RNHS. Our goal was to expand understanding of developmental skill trajectory with granular information on the proportion and timing of skill gain, loss, and regain on a larger set of developmental skills than previously characterized [13] to inform trial design and provide a natural history arm in novel disease modifying therapies for RTT.

These data are the most exhaustive description of developmental skill trajectory in RTT and are consistent with published findings [12, 13]. Broadly, we observed a greater percentage of individuals gaining rudimentary skills (acquired earlier in life) than more complex, later-acquired skills. For nearly all the skills assessed (50 of 51), the median age of acquisition (when gained) was under 2 years old, and for over half of the skills (26 of 51), 95% of individuals who gained a specific skill did so by 2 years old. Consistent with this early age of acquisition of skills, < 5% of individuals gained any skill after age 4 and < 2.5% after 6 years, indicating that gaining a new skill beyond these ages is highly unlikely.

While the age of diagnosis of RTT has decreased since the first description of the disorder in English [5, 12, 46, 53], the current median age of diagnosis (2.7 years) [46] is more than one year after both the previously reported median age of regression (18 months, 1.5 years) [12] and the median age of skill loss reported herein (Table 2). Comparing the age of skill acquisition to the expected age (based on CDC developmental milestone surveillance guidelines) [44] more than half of individuals with RTT did not acquire a specific skill by the CDC guideline threshold age. For nearly all CDC skills (39 of 41), > 20% of affected individuals did not obtain a specified skill. Importantly, a significant percentage of individuals with RTT do not obtain major developmental milestones by the age recommended by the CDC guidelines that should occur before the onset of regression (e.g. Pull to Stand, Walk Independently, Pincer Grasp, Words With Meaning). Furthermore, a significant percentage of people with RTT do not acquire many early developmental skills expected to be achieved before 6 months old (e.g. Lift Head, Hold Bottle). These observations have at least two relevant implications. First, if the current CDC surveillance guidelines were universally followed, it is likely that a larger number of individuals with RTT would be identified earlier. Second, it questions the concept that early skill development is “apparently normal” in RTT [54, 55], which has been questioned for nearly four decades (see comprehensive review [15]). Families often report that they had suspicion about their child’s development even within the first six months of life [12, 15, 56], and systematic retrospective analysis of early home videos of children subsequently diagnosed with RTT identified characteristic developmental abnormalities [15, 57–59]. Hence, earlier genetic testing upon first noticing lack of skill gains could facilitate earlier diagnosis. Development of potentially disease modifying therapies, such as gene therapy, could result in better outcomes with earlier identification. Ultimately, one might argue that newborn screening for pathogenic *MECP2* variants could provide rapid, early diagnosis of RTT enabling early interventions; however, this could carry significant risk as the mere presence of pathogenic *MECP2* variants is not sufficient to establish the clinical emergence of RTT [6, 60]. Together, early detection of alterations in early developmental milestones, paired with genetic testing, can lead to specific prediction of which individuals would benefit from early treatment with disease modifying therapies.

A general pattern emerged in our analysis of skill loss. More advanced and later acquired skills are lost in a larger percentage of individuals compared to loss of early developmental skills. For example, the frequency of loss of fine motor skills and expressive communication skills (especially verbal) was high, consistent with the fact that loss of hand skills and verbal communication are defining features of RTT and necessary criteria for the clinical diagnosis of RTT [6]. Most of the documented skill loss occurs within 2 years of initial gain. However, gross motor skill loss generally occurred over longer time intervals and at older ages than other skill domains (Fig. 2). This aligns with the concept of “Late Motor Decline” [7], evaluations focused on longitudinal gross motor performance [61, 62], and recognition of increasing rigidity and Parkinsonian features with age [10, 11]. The recognition that regression of most domains (aside from Gross Motor) typically occurs early in life and within the first 2 years after gain is important for counseling and discussion with caregivers. Similarly, acknowledging that loss of previously acquired gross motor skills may occur over a protracted time period and influenced by progressive motor tone abnormalities and Parkinsonian features underscores recommendations for medical interventions that modify tone and long-term physical therapies to maintain gross motor function.

The period after regression (Stage III, the “Pseudostationary” stage [7, 63]) historically has been characterized by the lack of further skill loss, although this has been noted to be invalid [7], and here we demonstrate that slow regression can occur in this stage. Additionally, reports of some degree of regaining lost skills during Stage III exist. While we observed that this can occur, it is rare (< 20% of individuals), predominantly in lower-level skills, and infrequent for higher-level skills. Further, regain of skills primarily occurs before age 6 years and within 2 years of loss. Thus, while regaining previously lost skills can occur in RTT, it is uncommon, occurs early in life, shortly after loss, and more frequently for lower-level skills.

Analysis of the one-year incidence of skill gain or regain demonstrated that individual skill gain or regain occurs early in life and drops off for nearly all skills by age 6 years. Furthermore, the one-year incidence of gaining or regaining at least one of the 51 skills assessed dropped below 10% after age 6 years. Overall, these results indicate that the trajectory of developmental skill gain, loss, and regain in RTT occurs early in life. After age 6 years it is unlikely that a skill not previously obtained will be gained or that a skill previously lost will be regained.

These results have implications for assessing therapeutic interventions, especially potentially disease-modifying interventions. The ability to describe those skills unlikely to be gained or regained as part of the natural history of RTT could be crucial in establishing reasonable trial outcomes to demonstrate improvement. This is especially critical for clinical trials that preclude conducting studies in a double-blind fashion. Such trials involving gene therapy require invasive procedures in children, utilize vector-based therapies with mortality and morbidity risks, require additional medications such as immunosuppressive agents that carry unacceptable risks in a placebo treated population, and could result in clinical features carrying the risk of unintentional unblinding. The advantages of these developmental endpoints are: 1) they are easily defined, 2) binary, and 3) likely to be emergent with an effective treatment paradigm. Current clinical trial outcome measures such as the Clinical Global Impression of Improvement (CGI-I) [39] or the Rett Syndrome Behavioral Questionnaire (RSBQ) [64–66] have limitations in terms of rater consistency (CGI-I) and limited coverage of important clinical features and sensitivity to change (RSBQ). The ability to apply these real-world natural history data to clinical trials offers a step forward. Importantly, a critical component that recognizes FDA guidance is the consideration of the “meaningfulness” of the gain or regain of a specific skill [67–73]. The impact of acquiring a skill on the overall quality of life for an affected individual, and their family, is likely not equivalent across the skills evaluated in this study. Gaining/regaining skills that impact adaptive activities, such as walking independently, self-feeding and self-care, and effectively communicating are likely to be more impactful than gaining/regaining lower-level skills such as lifting head or sitting with support. Thus, it is important to determine which skills would be most meaningful through input from those affected and their caregivers. As people with RTT have markedly impaired communication, input from caregivers on meaningfulness of specific skills is critical [67–73]. As demonstrated, limiting the set of developmental skills considered impacts the one-year incidence of either gaining or regaining any of the selected skills. The incorporation of caregiver input to define the most impactful skills, in combination with the expected trajectory of skill gain, loss, and regain, has robust potential to guide clinical trial design and implementation. Furthermore, the rich data provided from this study could be utilized to conduct “emulation” trials to define the expected trajectory and likelihood of gain or regain in an untreated cohort as a comparator to participants in an open label trial.

Although this work presents detailed information gathered from a large cohort of people with RTT, limitations are recognized. First, participants were primarily white, non-Hispanic participants in the US with sufficient resources to participate in this study [35]. While efforts were made to maximize participation and representation, the costs, associated loss of income, and difficulties of travel to examination sites could have significantly impacted the composition of the participants. This limits the extrapolation of these findings to other populations, including those outside of the US and from underrepresented populations. Second, the data collected on skill gain, loss, and regain was retrospectively collected from caregivers with the associated potential of recall bias. To mitigate this limitation, every effort was made to encourage families to refer to pictures, videos, baby books, medical records, or association with specific dates such as birthdays or holidays. Further, skill gain, loss, or regain was reviewed at every visit and considered in relation to the current ability of the participant. Because the occurrence of a skill event (gain, loss, or regain) is more likely to be remembered than the specific age of occurrence, we made no attempt to impute missing data for age of event when caregiver recall was not possible. Future studies could employ systematic evaluation of retrospectively captured video data from affected individuals [74] or prospectively captured longitudinal birth cohort studies [75], although the rare occurrence of RTT makes the latter challenging. Despite these limitations, the results presented here based on data collected from more than 1200 individuals with RTT through repeated, in-person assessments represents the largest body of work in this disorder and provides confidence in the reliability and representativeness of these findings. Future studies evaluating genotype/phenotype relationships and development of probabilistic models for the likelihood of individual or multiple skill gain/loss/regain will further expand our understanding of RTT.

## Conclusions

In summary, this comprehensive analysis demonstrates that the percentage of individuals with RTT who gain specific skills depends heavily on the developmental level of the skill, with higher-level abilities being acquired by fewer individuals. Skill gain, loss, and regain generally occur early in life, with a developmentally stable period expected after 4–6 years of age, except for gross motor skills which show a wider age range for skill loss. These natural history data provide fundamental information that can improve our understanding of developmental trajectories in RTT, facilitate earlier diagnosis, inform medical and therapeutic interventions, and guide clinical trial design in RTT.

## Supplementary Material

Supplementary Files

This is a list of supplementary files associated with this preprint. Click to download.

- AddFile1TableS1.pdf
- AddFile2TableS2.pdf
- AddFile3TableS3.pdf
- AddFile4SupplementalMethods.pdf
- AddFile5FigureS1.pdf
- AddFile6FigureS2.pdf
- AddFile7FigureS3.pdf
- AddFile8FigureS4.pdf
- AddFile9FigureS5.pdf

## Figures and Tables

**Table 1:** Developmental Skill Gain.

Skill Gain	All Cases		Age Gain (yr)		% Not Gained by	% Gain Beyond Age	
	Total N	% Gain (95% CI, N)	Mean (SEM, N)	Median (5%, 95%)	CDC (yr)	> 4yo (N)	> 6yo (N)
**Gross Motor**							
Lift Head	636	98.1 (96.7–99.0, 624)*	0.3 (0.02, 315)	0.3 (0.1, 0.8)	59.3 (0.2)	0.3 (1)	0.0 (0)
Roll From Tummy	1224	96.0 (94.7–97.0, 1175)*	0.5 (0.01, 983)	0.4 (0.2, 0.9)	25.9 (0.5)	0.0 (0)	0.0 (0)
Sit With Support	637	98.6 (97.3–99.4, 628)*	0.6 (0.02, 494)	0.5 (0.3, 0.9)	36.8 (0.5)	0.2 (1)	0.0 (0)
Sit Without Support	1227	97.1 (96.0–97.9, 1191)*	0.7 (0.01, 1050)	0.6 (0.3, 1.2)	14.6 (0.8)	0.4 (3)	0.3 (2)
Come To Sit	1227	83.0 (80.8–85.0, 1018)	0.9 (0.02, 843)	0.8 (0.4, 1.8)	45.1 (0.8)	0.3 (2)	0.1 (1)
Crawl	1225	70.9 (68.3–73.4, 868)	1.0 (0.02, 785)	0.9 (0.5, 1.8)	*N/A*	0.3 (3)	0.0 (0)
Stand With Support	633	85.6 (82.7–88.2, 542)	1.1 (0.03, 371)	1.0 (0.7, 2.0)	*N/A*	0.8 (3)	0.0 (0)
Pull To Stand	1228	67.7 (65–70.2, 831)	1.1 (0.02, 724)	1.0 (0.6, 2.0)	62.1 (10)	0.1 (1)	0.1 (1)
Walk With Support	1226	79.0 (76.7–81.3, 969)	1.4 (0.03, 850)	1.1 (0.8, 2.7)	66.6 (10)	1.4 (12)	0.3 (2)
Stand Independently	635	64.7 (61–68.4, 411)	1.6 (0.1, 261)	1.4 (0.8, 3.5)	*N/A*	1.7 (7)	0.5 (2)
Walk Independently	1227	59.3 (56.5–62.0, 727)	1.7 (0.04, 695)	1.5 (1.0, 3.3)	66.8 (15)	1.8 (17)	1.0 (8)
Ran 10 Feet	638	29.5 (26.0–33.1, 188)	1.9 (0.1, 99)	1.6 (1.1, 3.9)	86.5 (2.0)	0.5 (2)	0.5 (2)
Up Stairs With Help	636	53.9 (50.0–57.8, 343)	2.4 (0.1, 194)	2.0 (1.0, 5.0)	*N/A*	4.2 (17)	1.4 (5)
Up Stairs WO Help	1226	25.9 (23.5–28.4, 318)	2.3 (0.1, 235)	2.0 (1.0, 5.0)	87 (2.0)	1.9 (18)	0.5 (4)
Down Stairs With Help	637	44.0 (40.1–47.8, 280)	2.7 (0.1, 147)	2.0 (1.0, 6.0)	*N/A*	4.1 (16)	1.8 (6)
Down Stairs WO Help	1227	19.6 (17.5–21.9, 241)	2.7 (0.2, 173)	2.0 (1.0, 6.0)	*N/A*	2.1 (17)	1.3 (9)
**Fine Motor**							
Hold Bottle	1226	88.9 (87.1–90.6, 1090)	0.7 (0.01, 889)	0.6 (0.3, 1.3)	84.8 (0.3)	0.1 (1)	0.0 (0)
Reach For Toy	1226	97.6 (96.7–98.4, 1197)	0.6 (0.02, 934)	0.4 (0.2, 1.2)	32.2 (0.5)	0.4 (3)	0.3 (2)
Raking Grasp	636	89.8 (87.3–92.0, 571)	0.8 (0.1, 278)	0.6 (0.3, 2.0)	37 (0.8)	1.2 (3)	0.9 (2)
Transfer Objects	1226	76.8 (74.4–79.1, 942)	0.8 (0.03, 653)	0.7 (0.3, 2.0)	50.5 (0.8)	0.3 (2)	0.2 (1)
Pincer Grasp	1226	76.8 (74.3–79.1, 941)	1.0 (0.03, 708)	0.8 (0.5, 2.0)	41.6 (10)	0.9 (7)	0.4 (2)
Finger Feeding	1227	92.1 (90.4–93.5, 1130)*	0.9 (0.02, 915)	0.8 (0.5, 1.5)	16.7 (13)	0.4 (3)	0.2 (1)
Turn Pages in Book	636	56.0 (52.1–59.8, 356)	1.2 (0.1, 196)	1.0 (0.6, 2.5)	60.3 (2.5)	0.5 (2)	0.3 (1)
**Expressive**							
Social Smile	1225	99.5 (98.9–99.8, 1219)*	0.3 (0.01, 991)	0.2 (0.1, 0.8)	45.9 (0.2)	0.0 (0)	0.0 (0)
Cooing	1225	94.1 (92.7–95.4, 1153)*	0.3 (0.01, 861)	0.3 (0.1, 0.8)	24.4 (0.3)	0.1 (1)	0.0 (0)
Babbling	1224	95.3 (94.0–96.5, 1167)*	0.7 (0.02, 898)	0.5 (0.3, 1.3)	22.2 (0.8)	0.4 (3)	0.1 (1)
Words With Meaning	1226	76.7 (74.3–79.0, 940)	1.1 (0.02, 782)	1.0 (0.5, 2.0)	45.8 (10)	0.6 (5)	0.1 (1)
Spoken Phrases	1223	23.5 (21.1–25.9, 287)	2.0 (0.1, 196)	1.5 (1.0, 4.0)	86.9 (2.0)	2.0 (11)	1.4 (5)
Wave Bye	1224	58.5 (55.7–61.2, 716)	1.1 (0.1, 551)	0.8 (0.5, 2.6)	58.9 (10)	1.5 (10)	0.9 (5)
Point for Wants	1222	30.5 (28.0–33.1, 373)	1.7 (0.1, 265)	1.0 (0.5, 4.4)	84.2 (13)	2.2 (14)	1.6 (8)
Shared Stories	636	2.4 (1.4–3.7, 15)	6.9 (3.1, 6)	2.0 (1.3, 21)	99.5 (4.0)	1.0 (3)	0.9 (2)
**Receptive**							
Quiet To Voice	1223	90.0 (88.2–91.6, 1101)*	0.4 (0.03, 724)	0.2 (0.0, 1.5)	49.4 (0.2)	1.2 (4)	0.6 (3)
Respond To Sounds	637	97.5 (96.0–98.6, 621)*	0.3 (0.03, 311)	0.2 (0.0, 0.8)	39.6 (0.2)	0.3 (1)	0.0 (0)
Play Peek-A-Boo	633	62.6 (58.7–66.3, 396)	0.7 (0.04, 181)	0.6 (0.3, 1.5)	66.5 (0.8)	0.0 (0)	0.0 (0)
Respond To Words	636	91.7 (89.4–93.6, 583)	0.9 (0.1, 254)	0.7 (0.3, 2.0)	*N/A*	1.3 (2)	1.3 (2)
Respond To Own Name	637	96.2 (94.6–97.5, 613)	1.0 (0.1, 257)	0.7 (0.3, 3.0)	40.2 (0.8)	2.4 (4)	1.6 (2)
Inhibit To No	1225	72.4 (69.9–74.9, 887)	1.4 (0.1, 558)	1.0 (0.5, 3.5)	60.3 (10)	1.9 (13)	1.3 (8)
Commands With Gesture	1220	59.6 (56.8–62.3, 727)	1.8 (0.1, 460)	1.1 (0.5, 5.0)	71.7 (13)	4.5 (32)	2.2 (13)
Commands WO Gesture	1225	48.5 (45.7–51.3, 594)	2.0 (0.1, 366)	1.3 (0.7, 6.0)	77.7 (15)	4.6 (31)	2.4 (12)
Identify Body Parts	634	41.8 (38.0–45.7, 265)	2.4 (0.2, 120)	1.5 (0.8, 6.0)	82.4 (2.0)	3.2 (11)	2.0 (6)
Point to 1 Color	635	46.9 (43.1–50.8, 298)	3.9 (0.3, 134)	3.0 (1.0, 10.0)	88.1 (2.5)	12.3 (43)	7.3 (22)
**Social/Adaptive**							
Like Being Held	1223	96.0 (94.7–97.0, 1174)*	0.2 (0.02, 880)	0.1 (0.0, 0.7)	*N/A*	0.2 (2)	0.1 (1)
Attention to Sounds	634	89.4 (86.9–91.7, 567)	0.5 (0.1, 240)	0.3 (0.0, 1.3)	68.1 (0.2)	0.7 (2)	0.7 (2)
Eyes Fix and Follow	1221	95.1 (93.8–96.2, 1161)	0.5 (0.04, 749)	0.3 (0.1, 1.8)	70.2 (0.2)	1.4 (8)	1.1 (6)
Play Pat-A-Cake	633	28.1 (24.7–31.7, 178)	0.8 (0.05, 83)	0.8 (0.5, 1.6)	86.8 (10)	0.0 (0)	0.0 (0)
Desire Social Attention	632	84.8 (81.9–87.5, 536)	0.8 (0.1, 232)	0.3 (0.0, 3.0)	*N/A*	1.9 (4)	0.9 (1)
Imitate Peers	629	21.3 (18.2–24.6, 134)	2.2 (0.4, 58)	1.1 (0.7, 6.0)	94.2 (13)	1.6 (6)	1.0 (3)
Been Independent	630	35.6 (31.9–39.3, 224)	2.4 (0.3, 96)	1.3 (0.8, 8.0)	*N/A*	2.8 (9)	2.1 (5)
Drinks WO Help	636	61.5 (57.7–65.2, 391)	1.2 (0.1, 199)	1.0 (0.5, 2.5)	61.7 (15)	1.4 (5)	0.4 (1)
Uses Utensils With Help	636	67.5 (63.7–71.0, 429)	1.8 (0.1, 215)	1.2 (0.7, 5.0)	64.5 (15)	3.6 (13)	1.3 (4)
Uses Utensils WO Help	636	26.6 (23.2–30.1, 169)	2.3 (0.3, 76)	1.3 (0.8, 8.0)	88.4 (2.0)	2.3 (8)	1.9 (6)

The first column in the table displays the total number (N) for all cases with information available whether the specific developmental skill was gained or not. The second column shows the percentage (%), 95% Confidence Interval (CI) and number of individuals (N) who gained each skill.

* Indicates where the Clopper-Pearson exact method was used to calculate the CI. The third and fourth columns present the age of skill gain in years (yr) and the standard error of the mean (SEM) for cases with a known age of gain (N), and the median age of skill gain with 5–95% intervals yrs). The fifth column shows the percent of individuals who did not gain a specific skill by the CDC defined age threshold (displayed in parentheses). N/A indicates that no normative CDC threshold is available for that particular skill. Finally, the percentage of individuals who gain the skill beyond either 4 years old (yo) or 6 years old (yo) is displayed in the last two columns, including the total number (N) who gained the skill beyond the specific age cutoff. Skill domains are demarked on left side. Expressive Communication is labeled “Expressive” and Receptive Communication is labeled “Receptive”.

**Table 2:** Developmental Skill Loss.

Skill Loss	All Cases		Age Loss (yr)		Time Gain to Loss (yr)	
	Total N	% Loss (95% CI, N)	Mean (SEM, N)	Median (5%, 95%)	Mean (SEM, N)	Median (5%, 95%)
**Gross Motor**						
Lift Head	624	6.6 (4.8–8.8, 41)*	3.9 (1.2, 18)	1.6 (0.5, 14.0)	3.4 (1.4, 15)	1.2 (0.2, 13.9)
Roll From Tummy	1175	34.0 (31.4–36.8, 400)	3.6 (0.3, 281)	2.0 (0.7, 12.0)	2.9 (0.3, 255)	1.4 (0.1, 10.7)
Sit With Support	628	13.2 (10.7–16.0, 83)	5.2 (0.6, 61)	3.0 (1.0, 12.5)	4.6 (0.7, 53)	2.4 (0.2, 11.9)
Sit Without Support	1191	19.4 (17.2–21.7, 231)	5.1 (0.4, 179)	3.0 (0.9, 13.0)	4.4 (0.4, 170)	2.3 (0.2, 12.7)
Come To Sit	1018	26.8 (24.2–29.6, 273)	4.8 (0.3, 201)	3.0 (1.0, 13.0)	3.6 (0.3, 181)	1.7 (0.2, 12.2)
Crawl	868	25.3 (22.5–28.3, 220)	3.8 (0.3, 149)	2.0 (1.2, 12.0)	2.8 (0.3, 140)	1.2 (0.2, 11.1)
Stand With Support	542	28.4 (24.7–32.3, 154)	5.3 (0.6, 96)	2.2 (1.0, 17.5)	4.3 (0.7, 80)	1.2 (0.2, 18.2)
Pull To Stand	831	42.1 (38.8–45.5, 350)	4.1 (0.3, 266)	2.5 (1.3, 12.0)	2.9 (0.3, 253)	1.0 (0.1, 11.0)
Walk With Support	969	25.9 (23.2–28.7, 251)	4.8 (0.3, 188)	3.0 (1.2, 13.5)	3.5 (0.4, 172)	1.3 (0.1, 12.2)
Stand Independently	411	29.0 (24.7–33.5, 119)	7.8 (0.8, 74)	6.5 (1.0, 22.4)	6.6 (0.9, 58)	5.3 (0.1, 22.0)
Walk Independently	727	28.3 (25.1–31.7, 206)	7.8 (0.5, 177)	6.0 (1.3, 22.0)	6.0 (0.5, 164)	4.0 (0.0, 20.0)
Ran 10 Feet	188	42.6 (35.6–49.7, 80)	6.1 (0.8, 48)	4.0 (1.0, 16.0)	5.0 (1.1, 32)	2.3 (0.3, 14.8)
Up Stairs With Help	343	30.3 (25.6–35.3, 104)	7.3 (0.8, 55)	6.5 (1.4, 15.0)	4.4 (0.8, 43)	3.0 (0.3, 12.0)
Up Stairs Without Help	318	41.5 (36.2–47.0, 132)	4.7 (0.4, 96)	3.0 (1.4, 15.0)	2.8 (0.4, 81)	1.4 (0.2, 10.6)
Down Stairs With Help	280	31.1 (25.8–36.6, 87)	8.2 (0.9, 49)	7.3 (1.5, 22.0)	6.4 (1, 35)	5.0 (0.2, 17.0)
Down Stairs WO Help	241	44.0 (37.8–50.3, 106)	4.8 (0.5, 77)	3.0 (1.3, 14.0)	3.1 (0.5, 65)	1.0 (0.1, 12.5)
**Fine Motor**						
Hold Bottle	1090	60.5 (57.5–63.3, 659)	2.5 (0.1, 557)	2.0 (1.0, 6.0)	1.8 (0.1, 497)	1.2 (0.2, 5.0)
Reach For Toy	1197	56.8 (54.0–59.6, 680)	2.2 (0.1, 569)	1.7 (1.0, 4.5)	1.6 (0.1, 488)	1.2 (0.4, 3.7)
Raking Grasp	571	58.0 (53.9–62.0, 331)	2.8 (0.2, 219)	2.0 (1.0, 7.0)	2.2 (0.3, 148)	1.3 (0.3, 8.0)
Transfer Objects	942	71.0 (68.1–73.9, 669)	2.2 (0.1, 526)	1.8 (1.0, 4.5)	1.4 (0.1, 428)	1.0 (0.3, 3.5)
Pincer Grasp	941	76.2 (73.4–78.8, 717)	2.3 (0.1, 602)	2.0 (1.0, 5.0)	1.3 (0.1, 511)	1.0 (0.2, 3.4)
Finger Feeding	1130	63.7 (60.9–66.5, 720)	2.5 (0.1, 624)	2.0 (1.0, 5.8)	1.6 (0.1, 540)	1.0 (0.2, 4.2)
Turn Pages in Book	356	74.7 (70.0–79.0, 266)	2.4 (0.1, 156)	2.0 (1.2, 4.0)	1.3 (0.1, 113)	1.0 (0.3, 3.0)
**Expressive**						
Social Smile	1219	18.2 (16.1–20.4, 222)	2.2 (0.2, 200)	1.5 (0.8, 4.0)	1.6 (0.1, 178)	1.2 (0.3, 3.2)
Cooing	1153	40.2 (37.4–43.1, 464)	1.6 (0.1, 324)	1.3 (0.5, 3.0)	1.2 (0.1, 288)	1.0 (0.2, 2.6)
Babbling	1167	55.4 (52.6–58.3, 647)	1.9 (0.1, 518)	1.5 (0.8, 4.0)	1.1 (0.1, 459)	0.8 (0.2, 2.7)
Words With Meaning	940	88.6 (86.5–90.5, 833)	2.0 (0.1, 755)	1.5 (0.9, 4.0)	1.0 (0.1, 652)	0.5 (0.1, 2.5)
Spoken Phrases	287	87.8 (83.7–91.2, 252)	2.5 (0.1, 202)	2.0 (1.2, 5.0)	1.0 (0.1, 144)	0.5 (0.1, 2.5)
Wave Bye	716	87.8 (85.3–90.1, 629)	2.0 (0.1, 497)	1.5 (0.8, 4.0)	1.0 (0.1, 422)	0.7 (0.1, 2.5)
Point for Wants	373	67.3 (62.4–71.9, 251)	2.3 (0.2, 184)	1.9 (1.0, 4.0)	1.2 (0.2, 152)	0.7 (0.2, 3.0)
Shared Stories	15	66.7 (38.4–88.2, 10)*	2.7 (0.2, 6)	2.5 (2.0, 3.0)	1.1 (0.1, 3)	1.0 (1.0, 1.2)
**Receptive**						
Quiet To Voice	1101	23.7 (21.3–26.3, 261)	1.8 (0.1, 218)	1.5 (1.0, 3.5)	1.4 (0.1, 171)	1.2 (0.4, 2.9)
Respond To Sounds	621	15.8 (13.1–18.8, 98)	1.6 (0.1, 78)	1.5 (0.5, 3.0)	1.4 (0.1, 37)	1.2 (0.5, 2.9)
Play Peek-A-Boo	396	57.8 (52.9–62.6, 229)	1.9 (0.1, 111)	1.5 (0.8, 3.0)	1.1 (0.1, 87)	0.9 (0.2, 2.5)
Respond To Words	583	19.6 (16.5–22.9, 114)	1.9 (0.1, 73)	1.5 (1.0, 3.0)	1.1 (0.1, 55)	0.8 (0.3, 2.5)
Respond To Own Name	613	17.6 (14.7–20.8, 108)	1.7 (0.1, 75)	1.5 (0.8, 3.0)	0.9 (0.1, 52)	0.8 (0.2, 2.2)
Inhibit To No	887	26.0 (23.2–29.0, 231)	2.0 (0.1, 161)	1.6 (1.0, 4.0)	0.9 (0.1, 121)	0.7 (0.2, 2.1)
Commands With Gesture	727	48.1 (44.5–51.8, 350)	2.2 (0.1, 230)	1.9 (1.0, 5.0)	1.1 (0.1, 172)	0.8 (0.2, 3.0)
Commands WO Gesture	594	45.8 (41.8–49.8, 272)	2.4 (0.1, 171)	2.0 (1.1, 5.0)	1.2 (0.1, 128)	0.7 (0.2, 3.1)
Identify Body Parts	265	46.8 (40.8–52.8, 124)	2.5 (0.2, 68)	2.0 (1.1, 5.0)	1.1 (0.2, 55)	0.7 (0.2, 2.0)
Point to 1 Color	298	20.5 (16.2–25.3, 61)	2.2 (0.2, 28)	2.0 (0.3, 4.0)	0.7 (0.1, 21)	0.6 (0.2, 15)
**Social /Adaptive**						
Like Being Held	1174	19.5 (17.3–21.8, 229)	1.8 (0.1, 196)	1.5 (0.6, 3.5)	1.6 (0.1, 160)	1.4 (0.4, 2.9)
Attention to Loud Sounds	567	19.2 (16.1–22.6, 109)	1.8 (0.1, 80)	1.5 (1.0, 3.0)	1.1 (0.1, 29)	1.0 (0.2, 2.3)
Eyes Fix and Follow	1161	29.7 (27.1–32.4, 345)	1.7 (0.1, 278)	1.5 (0.8, 3.0)	1.3 (0, 214)	1.1 (0.4, 2.4)
Play Pat-A-Cake	178	78.7 (72.2–84.2, 140)	1.9 (0.1, 71)	1.5 (1.0, 3.0)	1.0 (0.1, 53)	0.7 (0.2, 2.2)
Desire Social Attention	536	17.9 (14.8–21.3, 96)	2.0 (0.2, 67)	1.5 (0.8, 4.0)	1.3 (0.2, 37)	1.1 (0.4, 2.0)
Imitate Peers	134	73.1 (65.2–80.2, 98)	2.4 (0.3, 66)	1.9 (0.9, 6.0)	1.5 (0.4, 35)	0.7 (0.2, 4.5)
Been Independent	224	42.0 (35.6–48.5, 94)	2.9 (0.4, 49)	2.0 (1.0, 7.0)	2.0 (0.6, 32)	0.8 (0.2, 4.0)
Drinks WO Help	391	62.4 (57.5–67.1, 244)	3.0 (0.3, 153)	2.0 (1.2, 6.4)	2.0 (0.3, 108)	1.0 (0.2, 6.3)
Uses Utensils With Help	429	52.9 (48.2–57.6, 227)	3.0 (0.2, 131)	2.0 (1.3, 7.0)	1.6 (0.2, 103)	1.0 (0.2, 5.0)
Uses Utensils WO Help	169	73.4 (66.4–79.7, 124)	3.0 (0.3, 71)	2.5 (1.3, 5.0)	1.2 (0.2, 50)	0.7 (0.2, 3.0)

The total number (N) of participants with information available on skill loss is shown in the first column. Column two displays the percentage (%), 95% Confidence Interval (CI) and number of individuals (N) who lost a previously gained skill for each skill for all cases.

* Indicates where the Clopper-Pearson exact method was used to calculate the CI. The mean age of skill loss in years (yr), with the standard error of the mean (SEM) and the median age of skill loss with 5–95% intervals are presented for cases with known age of loss. Similarly, for the time from gain to loss the mean, SEM, median and 5–95% intervals are shown for cases with known age of gain and loss.

**Table 3:** Developmental Skill Regain.

Skill Regain	Regain (All Cases)		Age Regain (yr)			Time Loss to Regain (yr)		
	Total N	% Regain (95% CI, N)	Mean (SEM, N)	Median (5%, 95%)	% Regain > 6 yo (N)	Mean (SEM, N)	Median (5%, 95%)	% Regain > 2yrs post loss (N)
**Gross Motor**								
Lift Head	41	36.6 (23–51.8, 15)	3.6 (0.7, 5)	3.0 (2.0, 6.0)	0.0 (0)	1.8 (0.6, 5)	1.4 (0.5, 4.0)	15.6 (2)
Roll From Tummy	400	14.0 (10.8–17.6, 56)	3.3 (0.4, 35)	2.7 (0.9, 7.3)	13 (4)	1.4 (0.3, 32)	0.8 (0.0, 3.5)	3.0 (7)
Sit With Support	83	31.3 (22.0–41.8, 26)	3.4 (0.6, 14)	3.0 (1.0, 7.0)	3.0 (2)	1.5 (0.4, 12)	1.0 (0.2, 3.3)	6.4 (3)
Sit Without Support	231	25.1 (19.8–31.0, 58)	5.1 (0.5, 43)	4.0 (1.6, 11.8)	7.0 (13)	2.3 (0.4, 41)	2.0 (0.2, 6.0)	10.7 (16)
Come To Sit	273	14.3 (10.5–18.8, 39)	4.9 (0.7, 29)	3.5 (1.3, 12.0)	3.9 (9)	2.0 (0.5, 28)	1.0 (0.0, 7.0)	5.4 (9)
Crawl	220	5.5 (2.8–9.3, 12)*	4.8 (1.1, 10)	2.5 (1.8, 12.0)	1.8 (3)	1.9 (0.6, 10)	1.1 (0.3, 6.0)	2.4 (3)
Stand With Support	154	11.7 (7.3–17.4, 18)	6.1 (1.4, 12)	4.0 (1.7, 13.0)	5.0 (6)	2.3 (0.8, 11)	1.0 (0.1, 5.7)	5.5 (4)
Pull To Stand	350	11.1 (8.1–14.7, 39)	4.2 (0.7, 23)	2.5 (1.6, 12.4)	15 (4)	1.3 (0.4, 22)	0.7 (0.1, 3.3)	14 (3)
Walk With Support	251	16.7 (12.5–21.7, 42)	5.5 (0.7, 32)	4.0 (1.8, 13.0)	5.2 (10)	2.0 (0.6, 31)	1.0 (0.1, 6.0)	4.4 (5)
Stand Independently	119	12.6 (7.5–19.3, 15)	4.9 (1.4, 8)	2.8 (1.7, 12.4)	3.1 (3)	0.9 (0.4, 8)	0.5 (0.1, 3.7)	17 (1)
Walk Independently	206	20.4 (15.3–26.2, 42)	5.5 (0.7, 30)	4.3 (1.3, 13.0)	6.8 (11)	0.9 (0.2, 28)	0.7 (0.0, 2.0)	0.7 (1)
Ran 10 Feet	80	8.8 (3.6–17.2, 7)*	4.8	4.0 (1.7, 12.4)	15 (1)	0.9 (0.3, 7)	0.8 (0.1, 2.2)	2.1 (1)
Up Stairs With Help	104	13.5 (7.8–20.9, 14)	8.0 (1.2, 9)	9.0 (3.0, 12.4)	7.3 (6)	3.9 (1.2, 9)	3.2 (0.1, 10.6)	15.9 (5)
Up Stairs Without Help	132	9.8 (5.3–16.3, 13)*	5.8 (1.1, 9)	5.5 (2.0, 12.0)	3.8 (4)	2.7 (1.4, 7)	0.5 (0.4, 10.6)	3.2 (2)
Down Stairs With Help	87	11.5 (5.9–19.3, 10)	7.8 (1.5, 6)	6.6 (3.0, 12.4)	5.6 (4)	3.6 (1.4, 5)	4.1 (0.1, 8.0)	10.3 (3)
Down Stairs WO Help	106	8.5 (4.0–15.5, 9)*	6.6 (0.8, 3)	6.6 (5.3, 8.0)	2.2 (2)	2.8 (0.7, 3)	2.3 (2.0, 4.1)	2.9 (2)
**Fine Motor**								
Hold Bottle	659	9.6 (7.4–12.1, 63)*	3.8 (0.4, 48)	3.1 (1.3, 10.0)	12 (5)	1.7 (0.3, 44)	1.0 (0.2, 4.8)	2.5 (11)
Reach For Toy	680	20.6 (17.7–23.7, 140)	3.4 (0.2, 97)	3.0 (1.5, 6.9)	15 (7)	1.7 (0.2, 96)	1.1 (0.2, 4.0)	4.8 (22)
Raking Grasp	331	9.4 (6.5–13.0, 31)*	3.2 (0.4, 19)	3.0 (1.3, 6.0)	0.4 (1)	1.5 (0.3, 18)	1.0 (0.2, 4.0)	16 (3)
Transfer Objects	669	5.5 (3.9–7.5, 37)*	4.1 (0.5, 28)	3.0 (1.5, 9.0)	0.9 (4)	1.8 (0.3, 27)	1.0 (0.0, 4.5)	1.8 (8)
Pincer Grasp	717	9.9 (7.8–12.3, 71)*	5.0 (0.4, 48)	4.0 (1.7, 12.0)	2.5 (12)	2.2 (0.3, 43)	1.5 (0.3, 6.0)	3.0 (14)
Finger Feeding	720	10.4 (8.3–12.8, 75)	4.0 (0.3, 53)	3.5 (1.7, 7.0)	11 (5)	1.6 (0.3, 51)	1.0 (0.2, 4.5)	2.2 (10)
Turn Pages in Book	266	5.3 (2.9–8.7, 14)*	4.3 (0.8, 6)	4.5 (2.0, 7.0)	0.4 (1)	1.9 (0.8, 5)	1.0 (0.4, 4.5)	1.5 (2)
**Expressive**								
Social Smile	222	80.6 (75.1–85.5, 179)	3.0 (0.2, 137)	2.5 (1.1, 6.0)	3.6 (4)	1.3 (0.2, 133)	0.9 (0.2, 3.5)	13.4 (19)
Cooing	464	21.6 (18–25.4, 100)	3.0 (0.3, 73)	2.5 (1.2, 6.0)	1.2 (2)	1.5 (0.3, 73)	1.0 (0.2, 3.3)	4.8 (11)
Babbling	647	28.6 (25.2–32.2, 185)	3.0 (0.2, 154)	2.5 (1.3, 6.5)	2.0 (9)	1.4 (0.2, 150)	0.9 (0.2, 4.0)	6.7 (28)
Words With Meaning	833	16.6 (14.1–19.2, 138)	3.5 (0.2, 119)	3.0 (1.5, 7.0)	17 (9)	1.6 (0.2, 114)	1.0 (0.2, 4.3)	3.5 (22)
Spoken Phrases	252	9.9 (6.5–14.3, 25)*	5.3 (0.7, 19)	4.3 (2.0, 9.0)	2.5 (5)	2.8 (0.7, 19)	1.5 (0.0, 6.0)	4.9 (8)
Wave Bye	629	7.0 (5.1–9.3, 44)*	3.2 (0.3, 30)	3.0 (1.5, 7.0)	0.6 (3)	1.7 (0.3, 26)	1.1 (0.2, 5.0)	1.0 (4)
Point for Wants	251	14.3 (10.4–19, 36)	4.6 (0.5, 27)	4.0 (2.0, 9.0)	2.1 (4)	2.7 (0.5, 26)	2.0 (0.5, 7.7)	7.3 (11)
Shared Stories	10	10.0 (0.3–44.5, 1)*	− (−, 0)	-	0.0 (0)	− (−, 0)	-	0.0 (0)
**Receptive**								
Quiet To Voice	261	66.7 (60.8–72.2, 174)	3.1 (0.2, 128)	2.7 (1.4, 6.0)	3.9 (6)	1.4 (0.1, 127)	1.0 (0.2, 3.0)	14.5 (24)
Respond To Sounds	98	79.6 (70.9–86.7, 78)	2.7 (0.2, 52)	2.0 (1.2, 5.0)	15 (1)	1.3 (0.2, 50)	1.0 (0.2, 3.5)	15.1 (9)
Play Peek-A-Boo	229	7.4 (4.4–11.6, 17)*	4.1 (0.6, 9)	4.5 (2.0, 8.0)	0.5 (1)	1.8 (0.5, 8)	1.5 (0.3, 3.5)	3.0 (3)
Respond To Words	114	52.6 (43.5–61.7, 60)	3.9 (0.6, 36)	3.0 (1.5, 8.0)	7.9 (5)	1.6 (0.3, 30)	1.0 (0.2, 5.0)	14.9 (8)
Respond To Own Name	108	72.2 (63.3–80.1, 78)	3.5 (0.4, 52)	2.8 (1.3, 6.8)	6.7 (4)	1.7 (0.4, 45)	1.0 (0.2, 4.5)	20.4 (11)
Inhibit To No	231	45.5 (39.1–51.9, 105)	3.9 (0.3, 62)	3.3 (1.5, 8.0)	4.5 (7)	2.0 (0.3, 58)	1.5 (0.3, 5.5)	12.0 (14)
Commands W Gesture	350	30.6 (25.9–35.5, 107)	4.1 (0.4, 55)	3.0 (1.6, 9.0)	3.5 (7)	1.9 (0.3, 54)	1.2 (0.2, 5.7)	5.6 (9)
Commands WO Gesture	272	31.6 (26.3–37.3, 86)	3.9 (0.3, 44)	3.3 (1.7, 7.0)	2.2 (4)	2.0 (0.3, 43)	1.5 (0.3, 5.7)	10.3 (13)
Identify Body Parts	124	14.5 (9.1–21.4, 18)	4.5 (0.7, 9)	3.5 (1.8, 8.0)	2.0 (2)	2.2 (0.5, 8)	2.0 (0.1, 4.0)	7.1 (4)
Point to 1 Color	61	32.8 (21.9–45.1, 20)	5.9 (1.3, 10)	5.0 (1.8, 15.0)	7.4 (3)	3.3 (1.1, 6)	1.7 (0.1, 7.5)	17.4 (2)
**Social/Adaptive**								
Like Being Held	229	73.4 (67.4–78.8, 168)	3.4 (0.2, 139)	3.0 (1.2, 7.0)	5.6 (9)	1.8 (0.2, 136)	1.1 (0.2, 4.2)	24.4 (41)
Attention to Sounds	109	68.8 (59.7–77, 75)	2.8 (0.2, 50)	2.0 (1.0, 6.5)	3.9 (3)	1.4 (0.2, 47)	1.0 (0.2, 4.0)	13.8 (9)
Eyes Fix and Follow	345	73.6 (68.8–78.1, 254)	3.3 (0.2, 191)	2.6 (1.4, 7.0)	7.4 (15)	1.5 (0.1, 182)	1.0 (0.2, 4.7)	16.0 (34)
Play Pat-A-Cake	140	2.1 (0.4–6.1, 3)*	6.5 (1.5, 2)	5.0 (5.0, 8.0)	0.8 (1)	3.5 (1.5, 2)	2.0 (2.0, 5.0)	17 (1)
Desire Social Attention	96	72.9 (63.5–81.1, 70)	3.0 (0.3, 44)	2.5 (1.2, 6.0)	3.8 (2)	1.3 (0.2, 41)	1.0 (0.1, 4.0)	11.3 (6)
Imitate Peers	98	11.2 (6.0–18.5, 11)	4.0 (0.5, 9)	4.0 (2.2, 6.6)	1.2 (1)	2.3 (0.4, 9)	2.0 (0.8, 4.3)	7.3 (4)
Been Independent	94	17.0 (10.4–25.5, 16)	5.7 (1.6, 11)	4.0 (2.0, 8.0)	6.5 (3)	2.3 (0.7, 8)	1.2 (0.3, 6.0)	8.1 (3)
Drinks WO Help	244	8.6 (5.4–12.9, 21)*	5.3 (1.1, 9)	4.5 (1.8, 12.0)	1.2 (2)	2.8 (1, 9)	1.5 (0.4, 8.5)	3.1 (3)
Uses Utensils W Help	227	12.8 (8.9–17.5, 29)	5.5 (0.7, 16)	4.5 (1.8, 9.0)	3.1 (5)	2.3 (0.6, 11)	1.5 (0.3, 4.5)	4.9 (5)
Uses Utensils WO Help	124	8.9 (4.5–15.3, 11)*	6.7 (1.5, 5)	8.0 (2.0, 10.0)	3.2 (3)	4.1 (1.3, 5)	5.5 (0.5, 7.0)	6.1 (3)

The total number (N) of participants with information available on skill regain is shown in the first column. Column two displays the total percentage (%), 95% Confidence Interval (CI) and number of individuals (N) who regained a previously lost skill for each skill for all cases.

* Indicates where the Clopper-Pearson exact method was used to calculate the CI. The mean age of skill regain in years (yr), with the standard error of the mean (SEM) and the median age of skill regain with 5–95% intervals is presented for cases with identified age of regain. The percentage of individuals (and number) who regained a skill after 6 years old (yo) is shown. Similarly, the mean, SEM, median, and 5–95% intervals for time from loss to regain in years (yr) is shown as well as the percentage of individuals (and number) who regained a skill more than 2 years after losing that skill is displayed for cases in which the age of loss and regain is known.

## Data Availability

The datasets from the Rett syndrome and Rett-related Disorders Natural History Study (RNHS) have been deposited to the database of Genotypes and Phenotypes (dbGAP) repository per a predefined schedule at regular intervals (https://www.ncbi.nlm.nih.gov/projects/gap/cgi-bin/study.cgi?study_id=phs000574.v1.p1). Additionally, the datasets from the RNHS were transferred to the International Rett Syndrome Foundation to facilitate access based on reasonable request to info@rettsyndrome.org, pursuant to any required data transfer and use agreements.

## Abbreviations

CDC: Centers for Disease Control
CGI-I: Clinical Global Impression of Improvement
CI: Confidence Interval
dpGAP: Database of Genotypes and Phenotypes
IQR: Interquartile Range
MECP2: Methyl-CpG-Binding Protein 2
N: Number
NICHD: National Institute of Child Health and Development
RNHS: Rett-related disorders Natural History Study
RSBQ: Rett Syndrome Behavioral Questionnaire
RTT: Rett syndrome
SEM: Standard Error of the Mean
